# Development of a therapy against metastatic bladder cancer using an interleukin-2 surface-modified MB49 bladder cancer stem cells vaccine

**DOI:** 10.1186/s13287-015-0211-1

**Published:** 2015-11-14

**Authors:** Yong-tong Zhu, Shi-yu Pang, Cheng-yong Lei, Yang Luo, Qing-jun Chu, Wan-long Tan

**Affiliations:** Department of Urology, Nanfang Hospital, Southern Medical University, Guangzhou, China; Center for Reproductive Medicine, Department of Obstetrics and Gynecology, Nanfang Hospital, Southern Medical University, Guangzhou, China

**Keywords:** Bladder cancer, MB49 cells, Cancer stem cells, Vaccine, Streptavidin- interleukin-2

## Abstract

**Introduction:**

In previous study the streptavidin interleukin-2 (SA-IL-2)-modified MB49 vaccine was effective against bladder cancer in a mouse model. However, a small portion of tumors regrew because the vaccine could not eliminate MB49 bladder cancer stem cells (MCSCs). Accordingly, we developed a SA-IL-2-modified MCSCs vaccine and evaluated its antitumor effects.

**Methods:**

MCSCs were isolated and identified in cancer stem cells (CSCs) characters, with high expression of CSCs markers, higher resistance to chemotherapy, greater migration in vitro, and stronger tumorigenicity in vivo. The SA-IL-2 MCSCs vaccine was prepared and its bioactivity was evaluated. The protective, therapeutic, specific and memory immune response in animal experiments were designed to identify whether the vaccine elicited antitumor immunity and acted against metastatic bladder cancer.

**Results:**

MCSCs had higher level of CD133 and CD44, less susceptibility to chemotherapy, more pronounced migration and greater tumorigenic ability. The successfully prepared SA-IL-2 MCSCs vaccine inhibited the tumor volume and prolonged mice survival in animal experiments. The expression of IgG, the population of dendritic cells, CD8^+^ and CD4^+^ T cells were highest in the experimental group than in the four control groups.

**Conclusions:**

The SA-IL-2 MCSCs vaccine induced an antitumor immune response and was used to eliminate MCSCs to prevent tumor regrowth.

## Introduction

Bladder cancer is the second most common urologic cancer after prostate cancer in the United States and the world [1]. For muscle invasive cancers, the standard treatment is radical cystectomy with pelvic lymphadenectomy. Nevertheless, more than 50 % of patients who undergo treatment will develop local or metastatic recurrence [2]. The human interleukin-2 (IL-2) surface modified MB49 bladder cancer cells vaccine induced specific antitumor immunity and was effective against metastatic bladder cancer in our previous study [3]. However, a small portion of the mouse bladder tumors underwent regression and regrew after a period of time because the cancer stem cells (CSCs) were not eliminated. Recurrence of solid tumors may be due to the inability of traditional chemotherapy and radiotherapy to eliminate CSCs [4]. The vaccine used in our previous study was not the CSCs vaccine and thus could not induce specific immunity directed against CSCs. In this study, MB49 bladder cancer stem cells (MCSCs) were successfully isolated by a modified approach based on a combination of limited dilution methods and serum-free culture medium (SFM) methods used in previous studies [5]. Thus, we developed a technology on the foundation of previous protein-anchor technology, produced a streptavidin mouse interleukin-2 (SA-IL-2)-modified MCSCs vaccine, and evaluated the antitumor effects of this vaccine in a MCSCs metastatic mouse model.

## Methods

### Establishment of MCSCs

MB49, a mouse bladder cancer cell line, was a gift from Dr. I. C. Summerhayes from the Lahey Clinic in Burlington, Massachusetts, USA. [3]. MCSCs were isolated from MB49 cells using a combination of limited dilution and SFM methods in our previous study [5].

### Identification of CSCs characters in MCSCs

MCSCs generated the next passages in 15 days. First, it is necessary to identify MCSCs in CSCs characters with high expression of CSCs markers, higher resistance to chemotherapy, greater migration in vitro, and stronger tumorigenicity in vivo.

#### Flow cytometry

MCSCs and MB49 cells were harvested separately, dissociated and labeled with fluorescein isothiocyanate (FITC) mouse antiCD44 (Miltenyi Biotec, Bergisch Gladbach, Germany) and phycoerythrin (PE) mouse anti-prominin-1 (Miltenyi Biotec). FITC rat IgG2b *κ* isotype control (eBioscience, San Diego, CA, USA) and PE rat IgG1 *κ* isotype control (eBioscience) were used as the negative control. The ratio of CD133^+^CD44^+^ cells was evaluated using a BD FACSAria cell sorter (Becton-Dickinson, San Jose, CA, USA).

#### Western blotting

The protein extracts were separated by electrophoresis and transferred to polyvinylidene difluoride membranes (Millipore, Billerica, MA, USA). Membranes were blocked and incubated using the primary antibody anti-CD133 (Abcam, Cambridge, MA, USA), anti-CD44 (Abcam) and anti-β-actin antibody (Abcam). Then membranes were incubated with anti-mouse secondary antibodies (Abcam). Finally, protein bands were detected using Fluor Chem FC2 (Alpha Innotech, San Leandro, CA, USA) and their intensity was analyzed using the Image Lab software.

#### Quantitative polymerase chain reaction

Total RNA was isolated using Arcturus PicoPure RNA isolation kit (Arcturus, Life Technologies, Union City, CA, USA). The RNA quality was verified using Bioanalyzer RNA Pico Chip (Agilent Technologies, Santa Clara, CA, USA). cDNAs were synthesized by reverse transcription using the Superscript III reverse transcriptase (Invitrogen, Union City, CA, USA). cDNAs were amplified using SYBR green PCR master mix (Bio-Rad, Hercules, CA, USA) on a 7500 real time PCR system (AB Applied Biosystems, Singapore). The sequences of the primers used are listed in Table 1. GAPDH was used as a negative control.

**Table 1:** Primers of selected genes

Gene name	Primers (forward/reverse)	Base pairs of product
CD133	F: 5′-CGGGATCCGAAAAACTGATCTGT-3′	615 bp
	R: 5′-CCGCTCGAGTTACCTAGTTACTCTCTCC-3′	
CD44	F: 5′-CCCTGCTACCAGAGACCAAGAC-3′	401 bp
	R; 5′-GCAGGTTCCTTGTCTCATCAGC-3′	
GAPDH	F: 5′-CCATGGAGAAGGCTGGGG-3′	198 bp
	R: 5′-CAAAGTTGTCATCCATGACC-3′	

#### Chemotherapy-resistance ability

The cells were seeded onto a 96-well plate at a density of 1 × 10^4^ per well. The chemotherapeutic agents paclitaxel (Sigma, Saint Louis, MO, USA) and cisplatin (Sigma) were added at different concentrations. After four days, CCK-8 was added and the absorbance value was recorded. Cell viability was calculated as the percentage points of the absorbance values in treated wells relative to untreated control wells.

#### Migratory ability in vitro

Cells were seeded, in pure RPMI1640 (1 × 10^4^ cells/0.25 ml/well), onto the upper well, and a 6.5-mm pore-size polycarbonate membrane chamber was inserted into the transwell apparatus (Costar, Cambridge, MA, USA). RPMI1640 containing 10 % fetal bovine serum (FBS) was added into the lower well. Cells were incubated and migrated to the bottom surface after 24 hours, fixed, stained, rinsed and examined by inverted microscopy.

#### Tumorigenic ability in vivo

All animal experiments performed were approved by the Ethics Committee of Southern Medical University under Contract 1116904. Cells were injected subcutaneously into four-week-old nude mice (Center of Experimental Animals, Southern Medical University, Guangzhou, China) at 1 × 10^6^ MB49 cells/mouse or 1 × 10^4^ MCSCs/mouse. The volume of the tumor xenograft was observed every week, removed at week 8 and measured.

### Preparation of SA-IL-2 MCSCs vaccine

#### Vaccine preparation

MCSCs were fixed in 30 % ethanol at room temperature for 30 minutes. Then ethanol-fixed MCSCs were incubated with EZ-Link Sulfo-NHS-LC-Biotin (Pierce Biotechnology, Rockford, IL, USA). The biotinylated cells were incubated with the SA-IL-2 fusion protein produced in our lab [3]. The final purified product was the SA-IL-2 MCSCs vaccine.

#### Evaluation of SA-IL-2 on the surface of MCSCs

Vaccine was labeled with FITC anti-IL-2 monoclonal antibody (BD Biosciences Pharmingen, San Diego, CA, USA) and evaluated using a BD FACSAria cell sorter. Biotinylated cells were used as the control group.

#### Bioactive assay of SA-IL-2 immobilized on the surface of MCSCs

After the vaccine was lysed, membrane fractions were harvested and suspended in complete medium. The SA-IL-2 bioactivity was evaluated through proliferation in bone marrow cells (BMCs), while the SA-green fluorescent protein (GFP) was used as the control. Membrane fractions and BMCs were incubated in 96-well plates, CCK-8 was added and the absorbance value was recorded as described previously.

#### Level of IL-2 on the vaccine

The level of IL-2 antibody on the vaccine was measured by Western blotting (WB) as described previously. The primary antibodies were anti- IL-2 (Abcam) and anti-beta II tubulin (Abcam), while SA-GFP was used for the control group.

### Animal experiments

Animal experiments were performed to ascertain whether the SA-IL-2 MCSCs vaccine elicited antitumor immunity and acted against metastatic bladder cancer. After establishing the mouse model, the experiments to investigate the protective, therapeutic, specific and memory immune responses were separately designed and conducted. During the therapeutic immune response experiment, some serum markers were specifically examined to study the mechanism of the SA-IL-2 MCSCs vaccine.

#### Lung metastasis and subcutaneous mouse model of MCSCs

C57BL/6 female mice were injected intravenously in the tail vein with 2 × 10^4^ MCSCs to establish a lung metastasis model. Mice were injected with 1 × 10^5^ MCSCs into the hind leg to establish a subcutaneous model.

#### Protective immune response experiment

All mice in the subcutaneous and pulmonary models were divided into five groups and every group consisted of 15 mice. The experimental group was given the SA-IL-2 MCSCs vaccine. The other groups received either ethanol-fixed MCSCs, SA-IL-2 MB49 cells vaccine, SA-IL-2, or phosphate-buffered saline (PBS).

Mice received the vaccines or other reagents in advance, and then were planted with MCSCs to observe the protective role of the vaccines. First, mice were inoculated with the SA-IL-2 MCSCs vaccine or other reagents subcutaneously on days 0, 4 and 8. Then mice received MCSCs on day 12 to establish the lung metastasis and the subcutaneous models as previously described. The survival time was recorded and the volume of subcutaneous tumors was measured.

#### Therapeutic immune response experiment

Mice were planted with MCSCs in advance, and then received the vaccine or other reagents to investigate the therapeutic role of vaccines. Mice were divided into five groups as mentioned above. First, the lung metastasis and subcutaneous model mice were established. Then, mice were injected with the SA-IL-2 MCSCs vaccines or other reagents on days 0, 4, 8 and 12. The survival rate and the volume of the subcutaneous tumors were measured.

#### Specific immune response experiment

Mice were planted with MCSCs and prostate cancer cells to investigate the specific role of the vaccines. On day 60 of the immunotherapy experiment, surviving or tumor-free mice were injected subcutaneously with RM-1 cells in the left hind leg and MCSCs in the right hind leg. The volume of the subcutaneous tumors was measured.

#### Memory immune response experiment

Mice were planted with MCSCs again to observe the role of vaccines on memory immunity. On day 60 of the protective and immunotherapy experiment, surviving mice in the lung metastasis experiment or tumor-free mice in the subcutaneous experiment were injected with 1 × 10^5^ MCSCs intravenously again. Blank mice were used as the control group, and survival time was recorded.

#### Tumor specific lymphocyte cytotoxicity assay

Splenocytes were isolated on day 19 and stimulated with the inactivated MCSCs plus hIL-2 (20 U/mL, R&D systems, Minneapolis, MN, USA) for five days. MCSCs and splenocytes were seeded onto 96-well plates, incubated and subsequently used as target and effector cells, separately. Lactate dehydrogenase activity was measured using the cytotox 96 non-radioactive cytotoxicity assay (Promega, Madison, WI, USA). The percentage of tumor specific cytotoxic T lymphocytes (CTL) was calculated as previously described [3].

#### ELISA for serum IgG antibodies

Blood was collected on day 19, congealed and the supernatant was harvested. The concentrations of IgG were measured using ELISA kits (Abcam) according to the manufacturer’s protocol. Optical density (OD) value was measured at 450 nm using a microplate reader.

#### Flow cytometry of dendritic cells

Splenocytes were isolated on day 19 and red blood cells were lysed. Splenocytes were labeled with PE anti-mCD11c (Biolegend, San Diego, CA. USA) and FITC anti-mCD80 (Biolegend). The ratio of CD11c^+^CD80^+^ cells was measured using a BD FACSAria cell sorter.

#### Flow cytometry of T cell subsets

Blood was collected on day 19 and stained with FITC anti-mCD8 (eBioscience) and PE anti-mCD4 (eBioscience). Then red blood cells were lysed and the ratio of CD8^+^ and CD4^+^ cells was measured using a BD FACSAria cell sorter.

### Statistical analysis

All analyses were performed by the SPSS19.0 software, setting significance at P < 0.05. Numeric data were expressed as the mean ± standard deviation and analyzed by Student’s t-test (between two groups) or one-way analysis of variance (ANOVA) (>2 groups). Survival rates were analyzed by the Kaplan-Meier method, and differences in survival between groups were analyzed by the log-rank test.

## Results

### Identification of MCSCs in CSCs characters

**Fig. 1:**
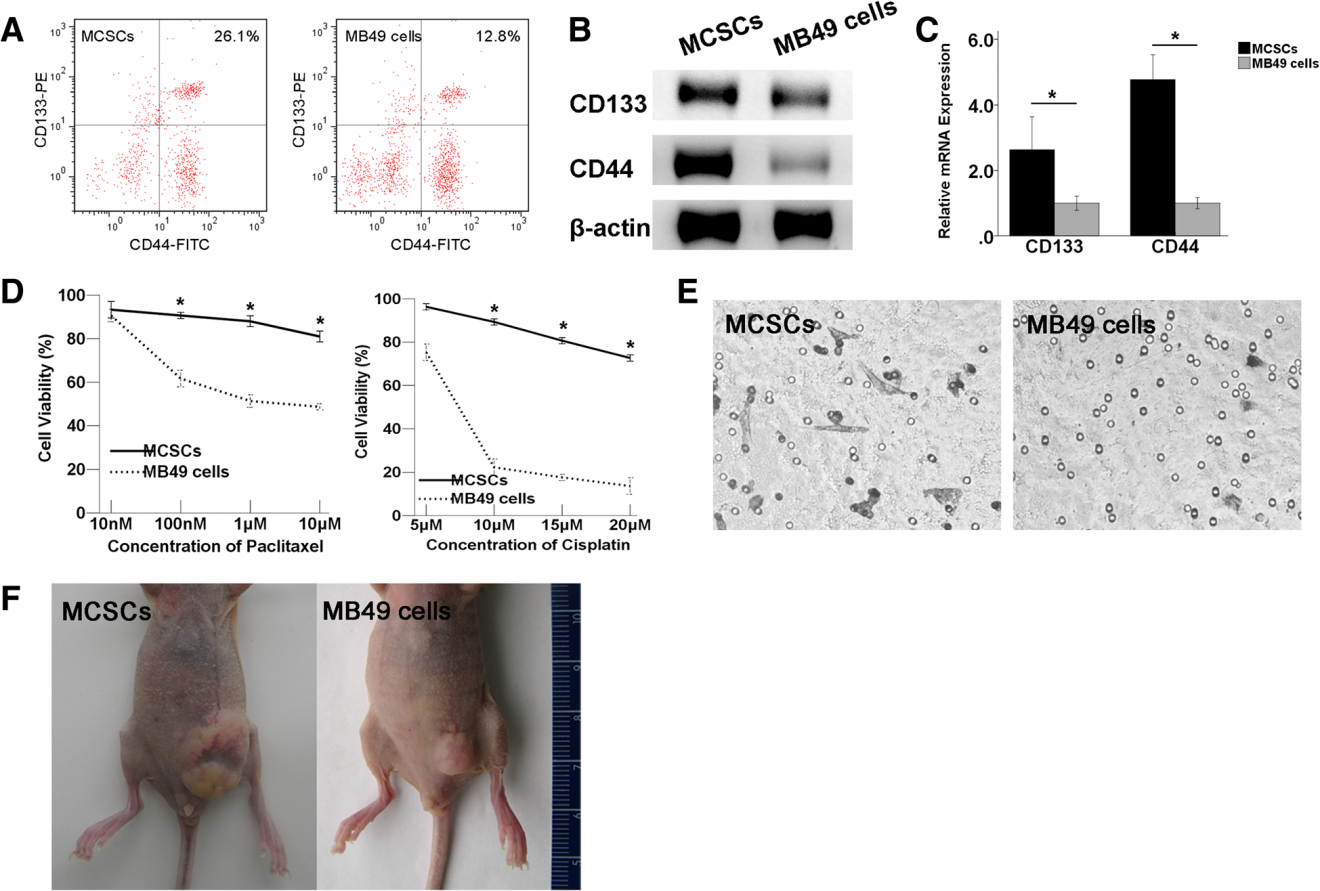
Identification of MCSCs in CSCs characters. **a** FCM analysis showed that the fraction of CD44^+^CD133^+^ cells in the MCSCs population was larger than in MB49 cells. **b** WB analysis showed that CD133 and CD44 were abundantly expressed in MCSCs but poorly expressed in MB49 cells. β-actin was used as a protein loading control. **c** qPCR analysis revealed that the relative levels of CD133 and CD44 mRNA in MCSCs were higher in MB49 cells. **d** MCSCs exhibited higher cell viabilities after being exposed to different concentrations of paclitaxel and cisplatin. **e** In the transwell migration assay, the number of invasive MCSCs was higher than that of MB49 cells. **f** In xenograft formation experiments, MCSCs produced larger tumor volumes than MB49 cells did. *P < 0.05 (vs MB49 cells). *MCSCs* MB49 bladder cancer stem cells, *CSCs* cancer stem cells, *FCM* flow cytometry, *WB* Western blot, *qPCR* quantitative polymerase chain reaction

Flow cytometry (FCM) analysis revealed that the fraction of CD44^+^CD133^+^ cells was 25.97±1.31 % in MCSCs and 12.70±0.66 % in MB49 cells (Fig. 1a). The WB analysis indicated that the CD133 and CD44 proteins were abundantly expressed in MCSCs, but much less in MB49 cells (Fig. 1b). The qPCR analysis showed that the relative levels of CD133 and CD44 mRNAs in MCSCs were 2.7 and 4.7 times higher, respectively, than those observed in MB49 cells (Fig. 1c).

Compared to MB49 cells, MCSCs displayed higher cell viabilities after being exposed to different concentrations of paclitaxel and cisplatin, which suggested that MCSCs had lower susceptibility to traditional anticancer agents (Fig. 1d).The results of the transwell migration assay indicated that more MCSCs invaded the bottom chamber when compared to MB49 cells under the same incubation conditions, which suggested that MCSCs had higher invasion ability than MB49 cells (Fig. 1e). Regarding xenograft formation, MCSCs produced tumors with larger volumes than MB49 cells did with the same number of injections (Fig. 1f).

### Preparation of SA-IL-2 MCSCs vaccine

**Fig. 2:**
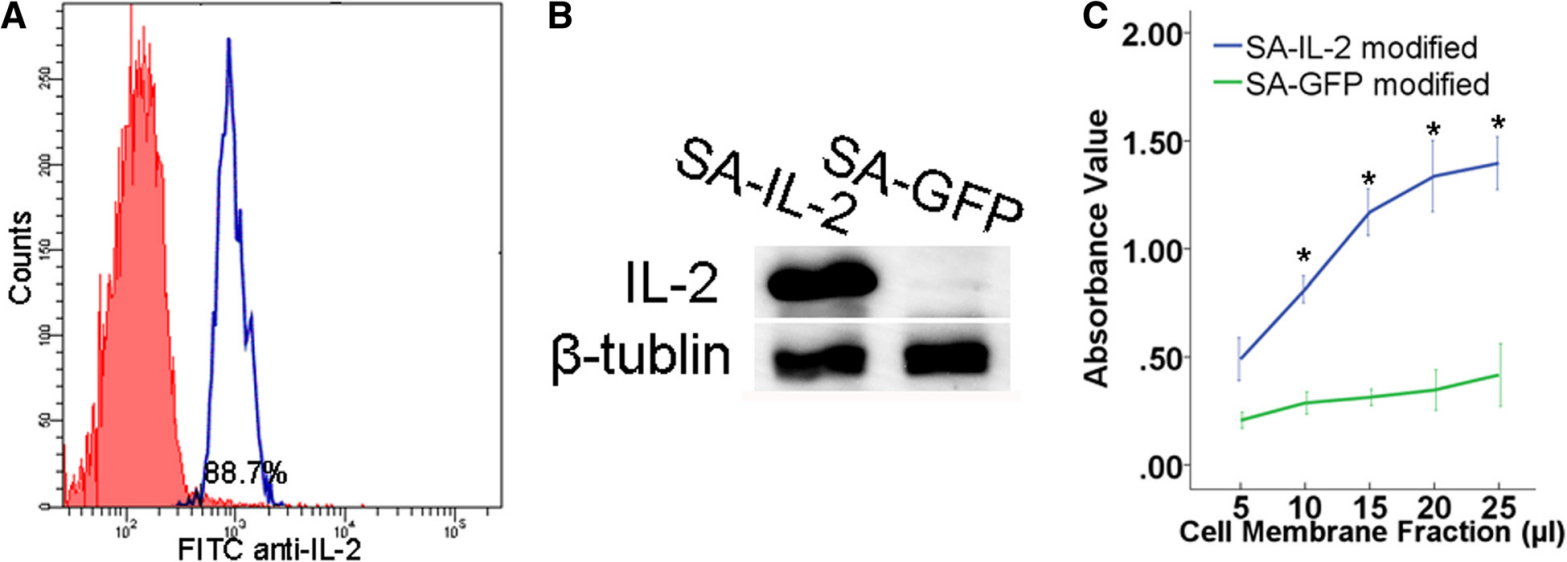
Analysis of the SA-IL-2 MCSCs vaccine. **a** Typical FCM analysis image of MCSCs anchored with SA-IL-2. **b** WB analysis showed that the IL-2 antibody was abundantly expressed on the vaccine. **c** CCK-8 assay showed that the proliferation of BMCs was stimulated by membrane bound IL-2 in a dosage dependent manner. SA-GFP was used as a control group. *P < 0.05 (vs control group) *SA-IL-2* streptavidin mouse interleukin-2, *MCSCs* MB49 bladder cancer stem cells, *WB* Western blot, *BMCs*, bone marrow cells, *GFP* green fluorescent protein

According to the results of the FCM analysis, the portion of MCSCs anchored with SA-IL-2 was 88.7 ± 1.1 % (Fig. 2a). Meanwhile, the WB analysis revealed that the IL-2 antibody was abundantly expressed on the vaccine (Fig. 2b). In addition, the CCK-8 assay results indicated that the proliferation of BMCs was stimulated by membrane bound IL-2 in a dosage dependent manner (Fig. 2c). These results showed that SA-IL-2 could be efficiently anchored on the outside of MCSCs and retained its biological activity well.

### Animal experiment

#### MCSCs vaccine induces a protective immune response

**Fig. 3:**
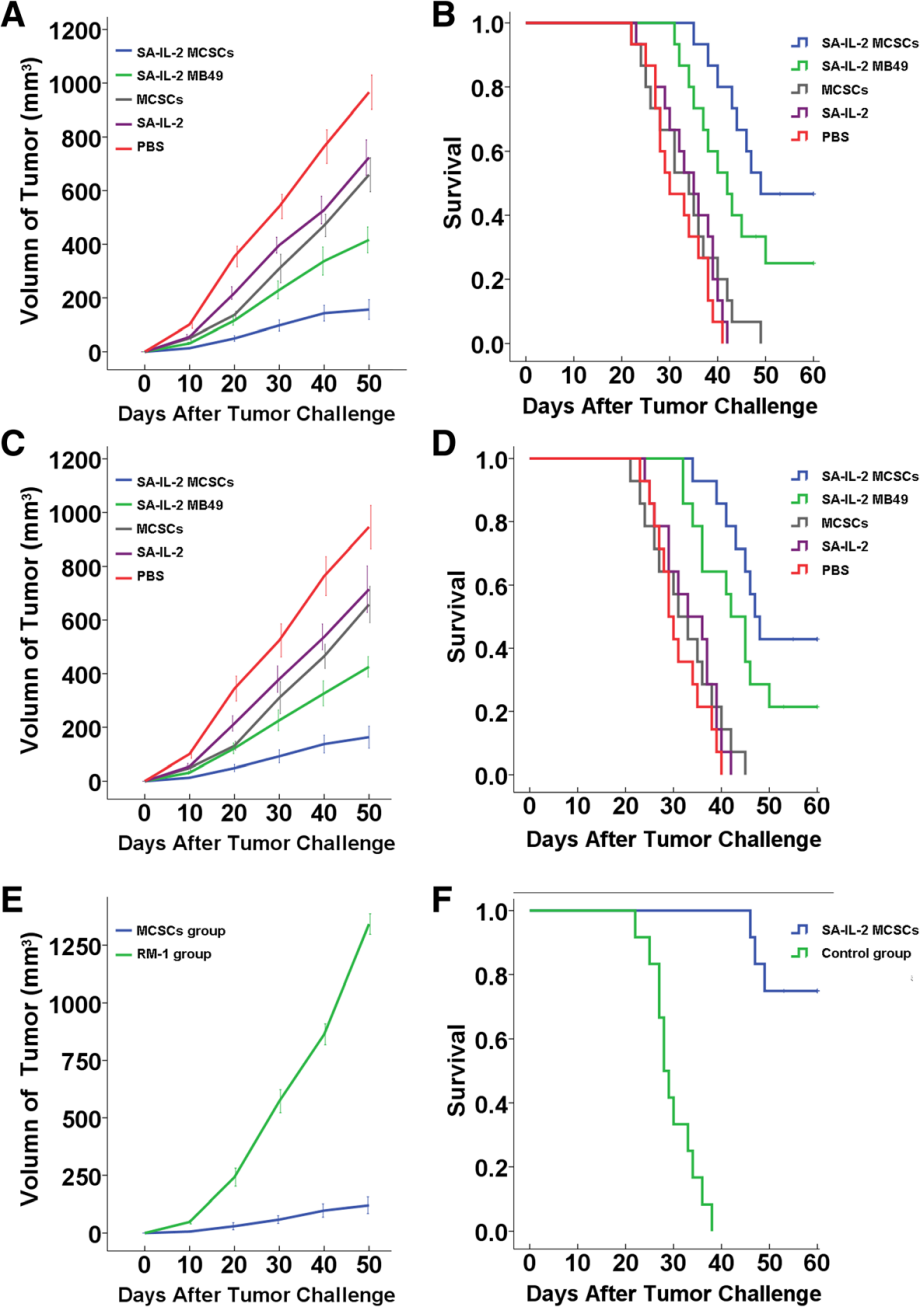
Animal experiment with the SA-IL-2 MCSCs vaccine. **a** In the protective experiment, experimental group mice exhibited a trend towards the smallest tumor volume. **b** In the protective experiment, mice in the experimental group had a longest survival. **c** In the therapeutic experiment, experimental group mice showed a trend towards the smallest tumor volume. **d** In the therapeutic experiment, experimental group mice had the longest survival. **e** In the specific experiment, experimental group mice showed a trend towards smaller tumor volume. **f** In the memory experiment, experimental mice group had a longer survival, *SA-IL-2* streptavidin mouse interleukin-2, *MCSCs* MB49 bladder cancer stem cells, *PBS* phosphate-buffered saline

In the subcutaneous model mice, the mean tumor volume in the experimental group was 156.3 mm^3^, and it exhibited a trend towards significantly smaller tumor volumes compared with the four control groups. Specifically, the mean tumor volumes in the groups receiving the SA-IL-2 MB49 cells vaccine, ethanol-fixed MCSCs, SA-IL-2, and PBS were 416.3, 659.3, 723.8, and 965.9 mm^3^, respectively (Fig. 3a).

In the pulmonary model mice, the survival rate in the experimental group was 50.8 days, and displayed a trend towards a significantly longer survival rate compared with the four control groups, whose survival rates were 44.3, 33.5, 33.9, and 31.7 days, respectively (Fig. 3b).

#### Therapeutic immune response experiment

In the subcutaneous model mice, the mean tumor volume in the experimental group was 163.1 mm^3^, and exhibited a trend towards significantly smaller tumor volumes compared with the four control groups. Specifically, the mean tumor volumes in the groups receiving the SA-IL-2 MB49 cells vaccine, ethanol-fixed MCSCs, SA-IL-2, and PBS were 425.5, 657.9, 714.9, and 946.0 mm^3^, respectively (Fig. 3c).

In the pulmonary model mice, the survival rate in the experimental group was 50.2 days, and exhibited a trend towards a significantly longer survival rate compared with the four control groups whose survival rates were 44.2, 32.2, 33.3, and 31.0 days, for the SA-IL-2 MB49 cells vaccine, ethanol-fixed MCSCs, SA-IL-2, and PBS groups, respectively (Fig. 3d).

#### Specific immune response with MCSCs vaccines

The mean tumor volume on the MCSCs injected side (120.4 mm^3^) was significantly smaller than that on the RM-1 cells injected side (1342.0 mm^3^), as shown in Fig. 3e. Such a result indicated that the SA-IL-2 vaccine could establish a firm tumor specific T cell immunity.

#### Memory immune response with MCSCs vaccines

After administering a second challenge of MCSCs, the survival rate in the experimental group (56.8 days) was significantly longer than that of the control group (29.8 days), as shown in Fig. 3f. Such a result demonstrated that the SA-IL-2 vaccine could produce long-term memory immunity.

#### Tumor-specific lymphocyte cytotoxicity assays

**Fig. 4:**
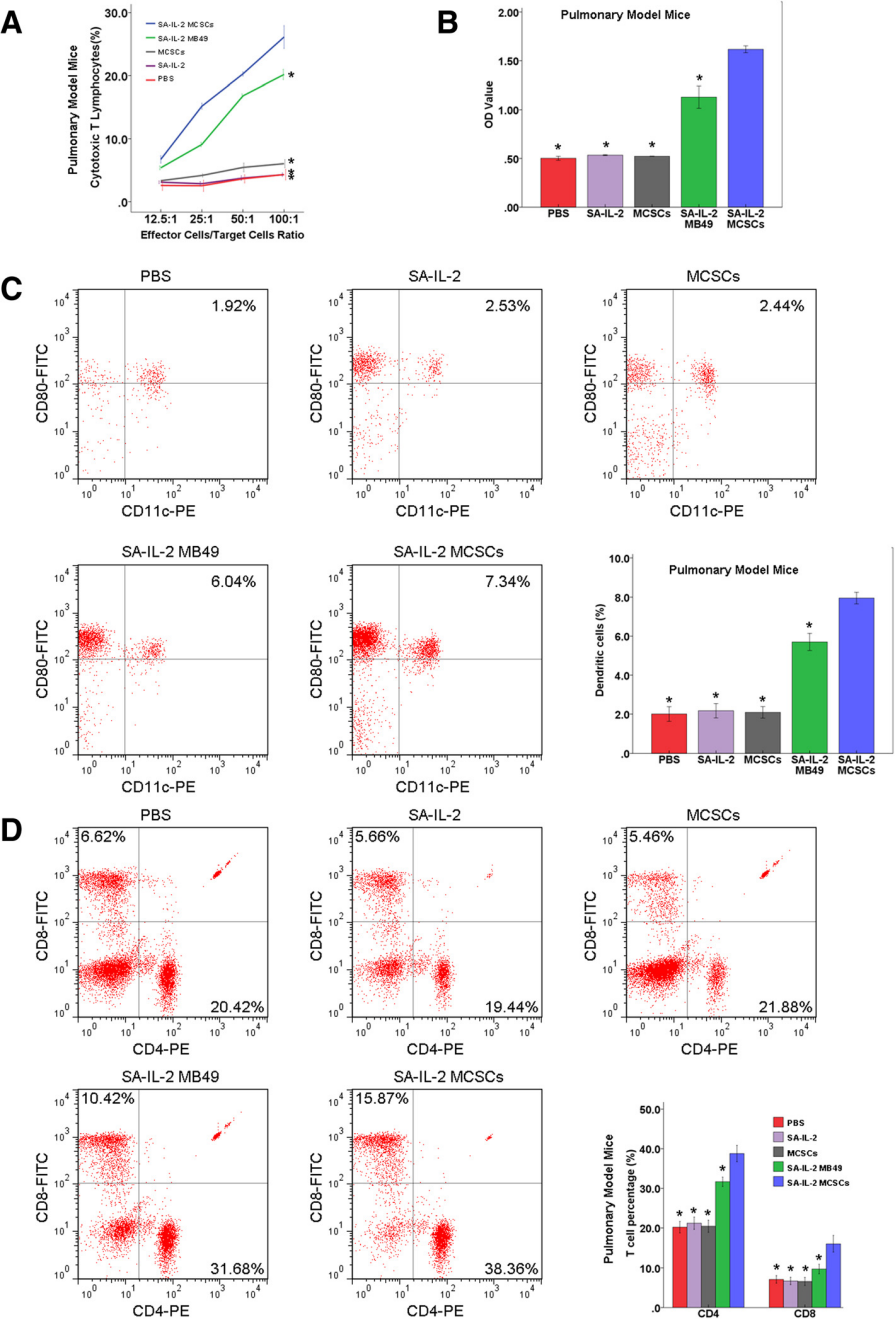
Antitumor potency of the SA-IL-2 MCSCs vaccine in the pulmonary model mice. **a** In the cytotoxicity assay, the portion of CTL in the experimental group was significantly higher than in the four control groups. **b** In ELISA assay, the expression of serum IgG antibodies in the experimental group was significantly higher than in the four control groups. **c** FCM analysis showed that the portion of DCs (CD11c^+^CD80^+^) in the experimental group was significantly larger than in the four control groups. **d** FCM analysis showed that the portion of CD8^+^and CD4^+^ T cells in the experimental group was significantly larger than in the four control groups. *P < 0.05 (vs experimental group). *SA-IL-2* streptavidin mouse interleukin-2, *MCSCs* MB49 bladder cancer stem cells, *CTL* cytotoxic T lymphocytes, *ELISA* enzyme-linked immunosorbent assay, *IgG* immunoglobulin G, *FCM* flow cytometry, *DCs* dendritic cells, *PBS* phosphate-buffered saline

**Fig. 5:**
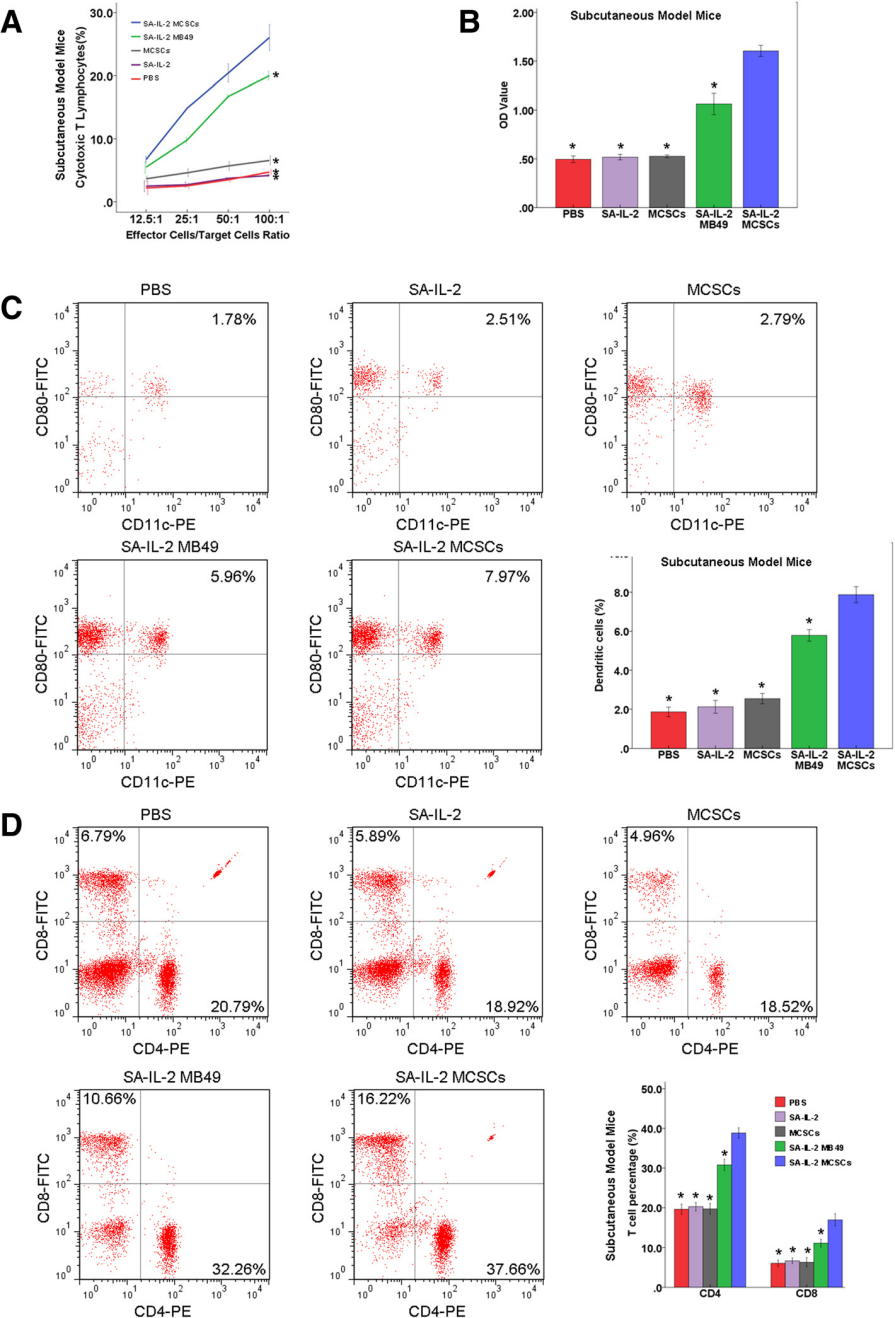
Antitumor potency of the SA-IL-2 MCSCs vaccine in the subcutaneous model mice. **a** In the cytotoxicity assay, the portion of CTL in the experimental group was significantly higher than in the four control groups. **b** In ELISA assay, the expression of serum IgG antibodies in the experimental group was significantly higher than in the four control groups. **c** FCM analysis showed that the portion of DCs (CD11c^+^CD80^+^) in the experimental group was significantly larger than in the four control groups. **d** FCM analysis showed that the portion of CD8^+^and CD4^+^ T cells in the experimental group was significantly larger than in the four control groups. *P < 0.05 (vs experimental group). *SA-IL-2* streptavidin mouse interleukin-2, *MCSCs* MB49 bladder cancer stem cells, *CTL* cytotoxic T lymphocytes, *ELISA* enzyme-linked immunosorbent assay, *IgG* immunoglobulin G, *FCM* flow cytometry, *DCs* dendritic cells, *PBS* phosphate-buffered saline

The portion of CTL was found to be significantly higher in the experimental group than in the four control groups (Figs. 4a and 5a). Such findings showed that the SA-IL-2 vaccine could establish a firmer tumor specific T cell immunity.

#### Detection of serum IgG antibodies

The expression of serum IgG antibodies in the experimental group was significantly higher than in the four control groups (Figs. 4b and 5b). Such an increased level of IgG could enhance the antitumor immunity effect in mice.

#### Number of DCs

According to the FCM analysis results, the portion of DCs (CD11c^+^CD80^+^) was significantly larger in the experimental group than in the four control groups (Figs. 4c and 5c). This result demonstrated that the SA-IL-2 vaccine could enhance the mature DCs population.

#### Number of T cell subsets

According to the findings of the FCM analysis, the portion of CD8^+^and CD4^+^ T cells in the experimental group was significantly larger than in the four control groups (Figs. 4d and 5d). Such an increased level of CD8^+^ and CD4^+^T lymphocytes could heighten the antitumor immunity effect in mice.

## Discussion

In this study an effective vaccine immunotherapy targeting CSCs was developed. To our knowledge, there has been little reported about therapy targeting the CSCs population in bladder cancer [6].

Similar to other methods [7] used to isolate CSCs, MCSCs isolated from MB49 cells in our previous study [5] were not 100 % pure of CSCs. The CD44 and CD133 markers were used to identify CSCs in tumor tissues [8, 9]. Additionally, our study found elevated expression levels of CD133^+^ and CD44^+^ in MCSCs (Fig. 1a-c). Targeting CD44^+^ and CD133^+^ cancer cells or pathways involving a CD133^+^CD44^+^ cell subpopulation might be a strategy for colorectal cancer therapy [10]. Thus CD133^+^CD44^+^ cells may be the enriched CSC subpopulation in MB49 bladder cancer cell populations. MCSCs used in experiments were enriched for CD133^+^ and CD44^+^ markers, but not seen to be 100 % dual positive cells by FCM analysis (Fig. 1a). In addition, the expression of both markers was found elevated in MCSCs not only at the mRNA expression (qPCR) level (Fig. 1c), but also at the protein expression (WB) level (Fig. 1b). There were other markers that have been used to identify CSCs from tumors, such as ABC transporters, aldehyde dehydrogenase, and so on. Detecting the status of these markers will help us to understand MCSCs in further research.

We functionally characterized the MCSCs populations by different techniques [11, 12]. Specifically, MCSCs had a greater ability to penetrate wells (Fig. 1e). Moreover, although chemotherapy killed most tumor cancer cells, it could not kill CSCs. Additionally, MCSCs exhibited a lower sensitivity to paclitaxel and cisplatin (Fig. 1d), which might be consistent with the theory of resistance to chemotherapy [13, 14]. Tumorigenicity in nude mice was the standard method used to evaluate the tumorigenic ability of CSCs [15]. MCSCs had a greater ability to form subcutaneous tumors in nude mice (Fig. 1f). Taking all the above results together, MCSCs showed specific CSC properties.

Although in previous study the SA-IL-2 MB49 cells vaccine induced antitumor immunity to MB49 cells and killed the tumor, it did not induce specific immunity to MCSCs. Accordingly, a small portion of the mice developed tumors again within a certain period of time because MCSCs were not eliminated. In order to eliminate MCSCs, the SA-IL-2 MCSCs vaccine was produced on the basis of previous vaccine [3], and the surface modification of the MCSCs vaccine was able to induce antitumor immunity to MCSCs.

Although the SA-IL-2 MB49 vaccine had effect on experimental group (MCSCs) than other control groups (ethanol-fixed MCSCs, SA-IL-2 and PBS). Compared to the original vaccine, the SA-IL-2 MCSCs vaccine efficiently inhibited the tumor growth and prolonged the survival of mice (Fig. 3a-d). Moreover, effector cells inhibited MCSCs growth in vitro in cytotoxicity assay, and mice were resistant to a second administration of the MCSCs after being successfully treated by the vaccine (Figs. 4a and 5a). As IgG accounts for more than 80 % of total Ig, the serum level of IgG reflects the level of total Ig. Our study detected an elevated higher serum IgG level in the experimental group than in the four control groups (Figs. 4b and 5b). Such a result indicated that the immunotherapy with the SA-IL-2 MCSCs vaccine could induce antitumor specific immunity against MCSCs.

In order to eliminate MCSCs, the current study using the SA-IL-2 MCSCs vaccine performed similar studies previously conducted with SA-IL-2 MB49 vaccine. Considering the role of the original vaccine, a combination using MCSCs vaccine and MB49 vaccine maybe a better treatment, as one targets the bladder cancer cells and the other targets the bladder CSCs for successful treatment of bladder cancer.

IL-2 could promote the transition of DCs from immature to mature forms, and plays an important role in the growth of DCs during immune response [16]. However, the impact of anticancer immunity depends on the function of T lymphocytes. DCs are the most effective antigen presenting cells and regulate T lymphocyte-mediated immunity [17]. MCSCs vaccine elicited an immune response mediated by T lymphocytes, such as the reaction of effector T cells targeting CSCs and the increase in CD8^+^ and CD4^+^ T cells [18]. Thus, CD8^+^ and CD4^+^ T lymphocyte cells are the primary effector cells in antitumor immunity. The vaccine containing CSCs antigens could lead to strong antitumor T cell immunity [19]. Therefore, IL-2, DCs and T lymphocytes are closely connected in the antitumor response. MCSCs can be identified and eliminated by CD8^+^ and CD4^+^ T lymphocytes, and the immunosuppressive effects of MCSCs can be overcome in mice tumor models.

There are some limitations that needed to be taken into account. During the animal experiments, it would be better to test the MCSCs vaccine on MB49 cells alone because MB49 cells also contained CD133^+^ and CD44^+^ dual positive cells. Considering that MCSCs had a higher level of CD133 and CD44, less susceptibility to chemotherapy, more pronounced migration and greater tumorigenic ability than MB49 cells, it was assumed that MCSCs vaccine alone would be effective to eliminate the entire population of MCSCs and MB49 cells. And the experiments that the mice injected with MB49 cells treated with the MCSCs vaccine are going to be recommended in the future. Furthermore, MCSCs were not 100 % pure of CSCs, so MCSCs vaccine did not induce 100 % tumor regression. MCSCs were able to give rise to MCSCs and MB49 cells, and generally CSCs were considered to be a rare population and eventually non-CSCs over populate the tumor. Although it was assumed that MCSCs vaccine would be effective to eliminate CSCs and non-CSCs, a better treatment using MCSCs vaccine and MB49 vaccine simultaneously is also recommended in the future.

## Conclusions

SA-IL-2 MCSCs vaccine was successfully produced and used to eliminate MCSCs to prevent tumor recurrence. However, the exact mechanism of the vaccine is still poorly understood. A better understanding of the functional aspects of the MCSCs vaccine could ultimately lead to clinical trials and eventually to its use to fight cancer in humans. A vaccine containing general cancer antigens or patient specific antigens may be the direction to follow for the development of a clinical vaccine in the future.

## Abbreviations

BMCs: bone marrow cells
CSCs: Cancer stem cells
CTLs: Cytotoxic T lymphocytes
DCs: Dendritic cells
ELISA: Enzyme-linked immunosorbent assay
FCM: Flow cytometry
FITC: Fluorescein isothiocyanate
GFP: Green fluorescent protein
IL-2: Interleukin-2
MCSCs: MB49 bladder cancer stem cells
qPCR: Quantitative polymerase chain reaction
SA-IL-2: Streptavidin mouse interleukin-2
WB: Western blotting
